# Myeloid differentiation protein 1 protected myocardial function against high‐fat stimulation induced pathological remodelling

**DOI:** 10.1111/jcmm.14407

**Published:** 2019-05-29

**Authors:** Cai‐Jie Shen, Bin Kong, Wei Shuai, Yu Liu, Guang‐Ji Wang, Min Xu, Jing‐Jing Zhao, Jin Fang, Hui Fu, Xiao‐Bo Jiang, He Huang

**Affiliations:** ^1^ Department of Cardiology Renmin Hospital of Wuhan University Wuhan PR China; ^2^ Cardiovascular Research Institute of Wuhan University Wuhan PR China; ^3^ Hubei Key Laboratory of Cardiology Wuhan PR China

**Keywords:** cardiac remodelling, free fatty acid, high‐fat diet, MAPK and NF‐κB signalling pathways, myeloid differentiation protein 1

## Abstract

Myeloid differentiation 1 (MD‐1) is a secreted protein that regulates the immune response of B cell through interacting with radioprotective 105 (RP105). Disrupted immune response may contribute to the development of cardiac diseases, while the roles of MD‐1 remain elusive. Our studies aimed to explore the functions and molecular mechanisms of MD‐1 in obesity‐induced cardiomyopathy. H9C2 myocardial cells were treated with free fatty acid (FFA) containing palmitic acid and oleic acid to challenge high‐fat stimulation and adenoviruses harbouring human MD‐1 coding sequences or shRNA for MD‐1 overexpression or knockdown in vitro. MD‐1 overexpression or knockdown transgenic mice were generated to assess the effects of MD‐1 on high‐fat diet (HD) induced cardiomyopathy in vivo. Our results showed that MD‐1 was down‐regulated in H9C2 cells exposed to FFA stimulation for 48 hours and in obesity mice induced by HD for 20 weeks. Both in vivo and in vitro*,* silencing of MD‐1 accelerated myocardial function injury induced by HD stimulation through increased cardiac hypertrophy and fibrosis, while overexpression of MD‐1 alleviated the effects of HD by inhibiting the process of cardiac remodelling. Moreover, the MAPK and NF‐κB pathways were overactivated in MD‐1 deficient mice and H9C2 cells after high‐fat treatment. Inhibition of MAPK and NF‐κB pathways played a cardioprotective role against the adverse effects of MD‐1 silencing on high‐fat stimulation induced pathological remodelling. In conclusion, MD‐1 protected myocardial function against high‐fat stimulation induced cardiac pathological remodelling through negative regulation for MAPK/NF‐κB signalling pathways, providing feasible strategies for obesity cardiomyopathy.

## 1 INTRODUCTION

Obesity is a metabolic disorder affecting millions of people worldwide. Patients with obesity are highly vulnerable to cardiovascular dysfunctions, including hypertrophic cardiomyopathy.^1^, ^2^ Featured with impaired diastolic function and left ventricular hypertrophy, obesity cardiomyopathy (OCM) patients exhibited disrupted substrate metabolism, oxidative stress, endoplasmic reticulum stress in the early stage.^2^, ^3^, ^4^, ^5^, ^6^, ^7^ These events cause the accumulation of lipid droplets in cardiomyocytes, changing the immune response and resulting in cardiac fibrosis, remodelling and compromised cardiac functions.^8^, ^9^, ^10^ In the process, immune system is vital in modulating the development of diabetic cardiomyopathy.^9^ However, the underlying mechanism is not fully understood.

Myeloid differentiation 1 (MD‐1) is a secreted glycoprotein produced by macrophages.^11^ It forms complex with radioprotective 105 (RP105), a Toll‐like receptor protein, to play critical roles in lipopolysaccharide (LPS) recognition by B cells.^11^, ^12^, ^13^ Recent studies have revealed that MD‐1/RP105 complex as a key regulator of high‐fat diet induced chronic inflammation in obesity.^14^, ^15^ Moreover, RP105 was proved to play cardioprotective function in against myocardial ischaemia‐reperfusion injury by suppressing TLP4 signalling pathways.^16^, ^17^ As an indispensable accessory molecule required for the cell surface expression of RP105,^18^ whether MD‐1 may also play essential roles in cardiac diseases needs to be further explored. Recently, Jiang et al reported that loss of MD1 exacerbated myocardial I/R injury and increased the susceptibility to ventricular arrhythmia, both of which are possibly related to the up‐regulation of TLR4/NF‐κB signalling pathway.^19^ Another study found that MD1 deficiency played an important role in accelerating the development of inflammatory atrial fibrosis and increasing vulnerability to AF in mice with HFD‐fed induced obesity.^20^ In our previous research, we have verified MD‐1 is a vital modulator of cardiac hypertrophy and fibrosis.^21^ Thus, we hypothesized that MD‐1 may also play vital effects in obesity‐induced cardiomyopathy.

In this study, utilizing free fatty acid (FFA) containing palmitic acid and oleic acid to mimic high‐fat environment in H9C2 cells, or high‐fat diet (HD) administration in vivo to induce hyperlipaemia cardiomyopathy mice and cell models. Results demonstrated that MD‐1 protected myocardial function against high‐fat stimulation induced cardiac pathological remodelling through negative regulation for MAPK/NF‐κB signalling pathways. Our studies are beneficial to better understand the mechanisms of obesity cardiomyopathy and provided a new strategy to treat obesity cardiomyopathy through targeting MD‐1.

## 2 MATERIALS AND METHODS

### 2.1 Reagents

Palmitic acid and oleic acid were purchased from Sigma Aldrich (USA). Inhibitors U0126 and Bay 11‐7082 were purchased from Selleck Chemicals (USA).

### 2.2 Cell culture and high‐fat stimulation

Rat cardiomyocytes H9C2 cells were purchased from ATCC and cultured in Dulbecco's modified essential medium (DMEM) supplemented with 10% foetal bovine serum (FBS), 2 mmol/L glutamine, 1 mmol/L pyruvate, 100 U/mL penicillin and 100 mg/mL streptomycin. Cells were maintained in a humidified incubator with 5% CO_2_ at 37°C.

Free fatty acid (FFA), containing oleic acid and palmitic acid at 2:1 molar ratio, was prepared with fat‐free bovine serum albumin (BSA) as previously described 22, 23 and then added into the complete medium with final concentration of 0.25, 0.5 and 1 mmol/L. The control group was treated with 1% BSA. After treated with FFA for 16, 24 or 48 hours, cells were collected for analysis.

### 2.3 MTT assay

H9C2 cells were seeded on 96‐well microplates at 1 × 10^4^ cells/well and were treated with FFA in different concentration and time. Then 20 μL of MTT assay solution was added and incubated with cells for 4 hours. At the end of the incubation, plates were measured at 570 nm using a microplate reader (Molecular Devices, Menlo Park, CA). The absorbance value was positively correlated with the number of cells, to a certain extent, reflecting the proliferation ability of cells.

### 2.4 Oil red staining

The lipid deposition within the cells was assessed by oil red O staining. H9C2 cells were fixed with pre‐cooled 4% formaldehyde. Then cells were stained with oil red O (Sigma‐Aldrich) and counterstained with hematoxylin to visualize lipid vacuoles. Images were photographed with microscope (Olympus, Tokyo, Japan).

### 2.5 Construction and infection of the recombinant adenovirus

The full coding region of the human MD‐1 gene controlled by the cytomegalovirus (CMV) promoter was cloned into a replication‐defective adenoviral vector (Ad‐MD‐1). Parallelly, adenoviral vector containing GFP gene (Ad‐GFP) was used as control. Short hairpin RNAs (shRNAs) for MD‐1 knockdown and the negative control shRNA (shRNA‐NC) constructs purchased from GenePharma (Shanghai, China) were used to generate MD‐1 knockdown adenovirus (Ad‐shMD‐1) and control (Ad‐shNC) respectively. After packaging, amplification and purification, the viral titres were measured by a plaque assay with fluorescence counting in HEK‐293 cells. Cardiomyocyte H9C2 cells were infected with these adenoviruses at a multiplicity of infection (MOI) of 100.

### 2.6 RNA extraction and quantification polymerase chain reaction

Total RNA was extracted from H9C2 and mouse tissues using Trizol Reagent (Invitrogen, Paisley, UK) and cDNA was synthesized by reverse transcription using PrimeScript 1st Strand cDNA Synthesis Kit (Takara). The gene expression was measured by quantification polymerase chain reaction (qPCR) with Applied Biosystems 7500 Fast Real Time qPCR machine. The relative expression levels were calculated using 2^−∆∆Ct^ method, and GAPDH was used as an internal reference. The sequences of primers used for qPCR were listed in Table 1.

**Table 1:** Paired primer sequences used in qPCR

Genes	Paired primers	Sequences (5′‐3′)
MD‐1	Sense	CGGAGGCTTGGAAGTAGTCT
	Antisense	CGCCACAAAAGCTATGTCCA
ANP	Sense	ATTGACAGGATTGGAGCCCA
	Antisense	CAGAGTGGGAGAGGTAAGGC
BNP	Sense	AGTCCTAGCCAGTCTCCAGA
	Antisense	GTCTCTCCTGGATCCGGAAG
β‐MHC	Sense	GGAGGAGATCAGTGAGAGGC
	Antisense	GCTTCACCCGCTGTAGATTG
Col1a1	Sense	AACAAGGGAGGAGAGAGTGC
	Antisense	AGTCTCTTGCTTCCTCCCAC
Col3a1	Sense	GAAGGGCAGGGAACAACTGA
	Antisense	GGGCAGTCTAGTGGCTCATC
CTGF	Sense	CTGTGGGAGAAAACACCCCA
	Antisense	CACTCTTCCAGGAGGCTCAC
GAPDH	Sense	CCAGGTGGTCTCCTCTGA
	Antisense	GCTGTAGCCAAATCGTTGT

### 2.7 Tetraethyl rhodamine isothiocyanate‐phalloidin staining

H9C2 cells were grown on glass coverslips and treated with FFA or BSA. At the end of treatment, cells were washed and fixed by 4% paraformaldehyde for 15 minutes at room temperature. After washed in PBS, the cells were permeabilized in 0.5% (v/v) Triton X‐100 in PBS, washed and then stained with tetraethyl rhodamine isothiocyanate (TRITC, rhodamine)‐labelled phalloidin (1:200, Invitrogen, USA) in PBS and DAPI (1:1000, Beyotime, China) was added. All slides were washed and mounted. Slides were examined by a confocal laser scanning microscope (Olympus, Japan).

### 2.8 Western blot

Total protein was extracted and separated on 8%‐10% SDS‐PAGE. Then proteins were transferred onto PVDF membranes (EMD Millipore, Billerica, MA), and blocked with 5% BSA for 1 hour at room temperature. The membranes were subsequently incubated overnight at 4°C with the primary antibodies. Primary antibody for MD‐1 were purchased from Santa Cruz Biotechnology (sc‐390613, CA), and primary antibodies for p‐MEK (#9154), MEK (#9122), p‐ERK1/2 (#4370), ERK1/2 (#4695), p‐IκBα (#9246), IκBα (#4814), p‐NF‐κB p65 (#3033), NF‐κB p65 (#8242), p‐JNK1/2 (#4668), JNK1/2 (#9252), p‐p38 (#4511), p38 (#9212) and GAPDH (#5174) used for internal reference were purchased from Cell Signaling Technology (Danvers, MA). The membranes were washed and then incubated with HRP‐conjugated secondary antibody (1:5000) for 1 hour. Finally, the membranes were washed and exposed to ECL (Millipore) substrate and visualized using the chemiluminescence detection system (Bio‐Rad). The intensity of the bands was quantified using Image J software tools.

### 2.9 MD‐1 overexpression or knockdown transgenic mice

The MD‐1 deficient mice (MD‐1^−/−^) were purchased from Japan RIKEN Bioresource Centre Mouse (BRC) (B6.129P2‐MD‐1<tm1Kmiy>). To generate MD‐1 overexpression mice, the full‐length human MD‐1 cDNA was cloned to the downstream of the human cardiac α‐MHC promoter. Then the α‐MHC‐MD‐1 construct was microinjected into fertilized mouse embryos (C57BL/6 background). These transgenic mice were validated with PCR and western blot analysis. All these mice were raised under standard conditions with 12 hours/12 hours light‐dark cycle, 50 ± 15% humidity and 22 ± 2°C temperature. Before 6‐week‐old, all mice were fed with standard diet (ND, 10% kcal from fat) freely. From 6‐week‐old, some mice were fed with high‐fat diet (HD, 60% kcal from fat, Beijing HFK Bio‐Technology Co. LTD, no.H10060) for 20 weeks according to groups (n = 12).

All protocols were approved by the Animal Care and Use Committee of Renmin Hospital of Wuhan University and conducted in accordance with the Guide for the Care and Use of Laboratory Animals published by the US National Institutes of Health (NIH Publication No. 85‐23, revised 1996).

### 2.10 Glucose tolerance testing

Two days before end of the 20th week of HD feed, a glucose tolerance test (GTT) was performed on all these mice. Briefly, all mice were subjected to an overnight fast (≈16 hours) before test. Glucose at dose of 2 g/kg·body‐weight was intraperitoneally injected into the mice. At 0 minute before glucose injection and at 15, 30, 60, 90 and 120 minutes after glucose injection, the blood glucose was measured, respectively, using a glucometer (OneTouch Ultra, Johnson & Johnson, USA) through collecting blood from tail vein.

### 2.11 Echocardiography and serum lipid index analysis

At the end of treatment, mice were anesthetized by intraperitoneally injecting with 3% pentobarbital sodium at a dose of 30 mg/kg. Half hour later, the mice were fixed on the test plate, removed the hairs and coated with conductive fluid on the heart, then echocardiography analysis was performed using an ultrasonic apparatus Mylab30CV (Esaote SpA) instrument with a 10 MHz linear array ultrasound transducer according to the manufacturer's introduction and as previously described.^21^ The measurements including heart rate (HR), left ventricular end‐diastolic diameter (LVDd), left ventricular end‐systolic diameter (LVDs), diastolic interventricular septum (IVSd), systolic interventricular septum (IVSs) and fractional shortening (FS) were analysed by matched software.

After echocardiography experiments, mice were sacrificed. Serum was collected for biochemical determination including total cholesterol (TC, mmol/L), triglyceride (mmol/L), low density lipoprotein (LDL, mmol/L) and creatine kinase‐MB (CK‐MB, U/L). The hearts were dissected and weighed to compare heart weight (HW, g), body weight (BW, g), the ratios of HW/BW (%) and heart weight to tibia length (HW/TL, mg/mm).

### 2.12 Masson's trichrome staining and immunohistochemistry

Paraffin‐embedded sections from heart tissues were stained with Masson's trichrome to determine the degree of cardiac fibrosis. Masson's trichrome staining kit was purchased from Sigma Aldrich (MO, USA) and sections were stained according to the manufacturer's instruction.

For immunohistochemistry, the slices were deparaffinized, hydrated and treated for antigen retrieval, and then incubated with NF‐κB p65 primary antibody (1:500 dilution, Abcam, USA) at 4°C for overnight. The positive reactions were visualized as brown using a DAB (3, 3‐diamino‐benzidine) kit (Sigma, USA) and haematoxylin for nuclear counterstaining.

### 2.13 Statistics analysis

Data were presented as mean ± Standard deviation (SD) based on at least three different determinations. Student's *t* test was employed to compare the difference between two groups. The statistical analysis between multi‐groups was carried out using one‐way analysis of variance (ANOVA) by Tukey's post hoc test. A two‐side value of *P* < 0.05 was considered statistically significant. All statistical analyses were performed by GraphPad Prism 5 (GraphPad Software, La Jolla, CA).

## 3 RESULTS

### 3.1 FFA induces lipid accumulation and MD‐1 differential expression in cardiomyocytes

**Figure 1:**
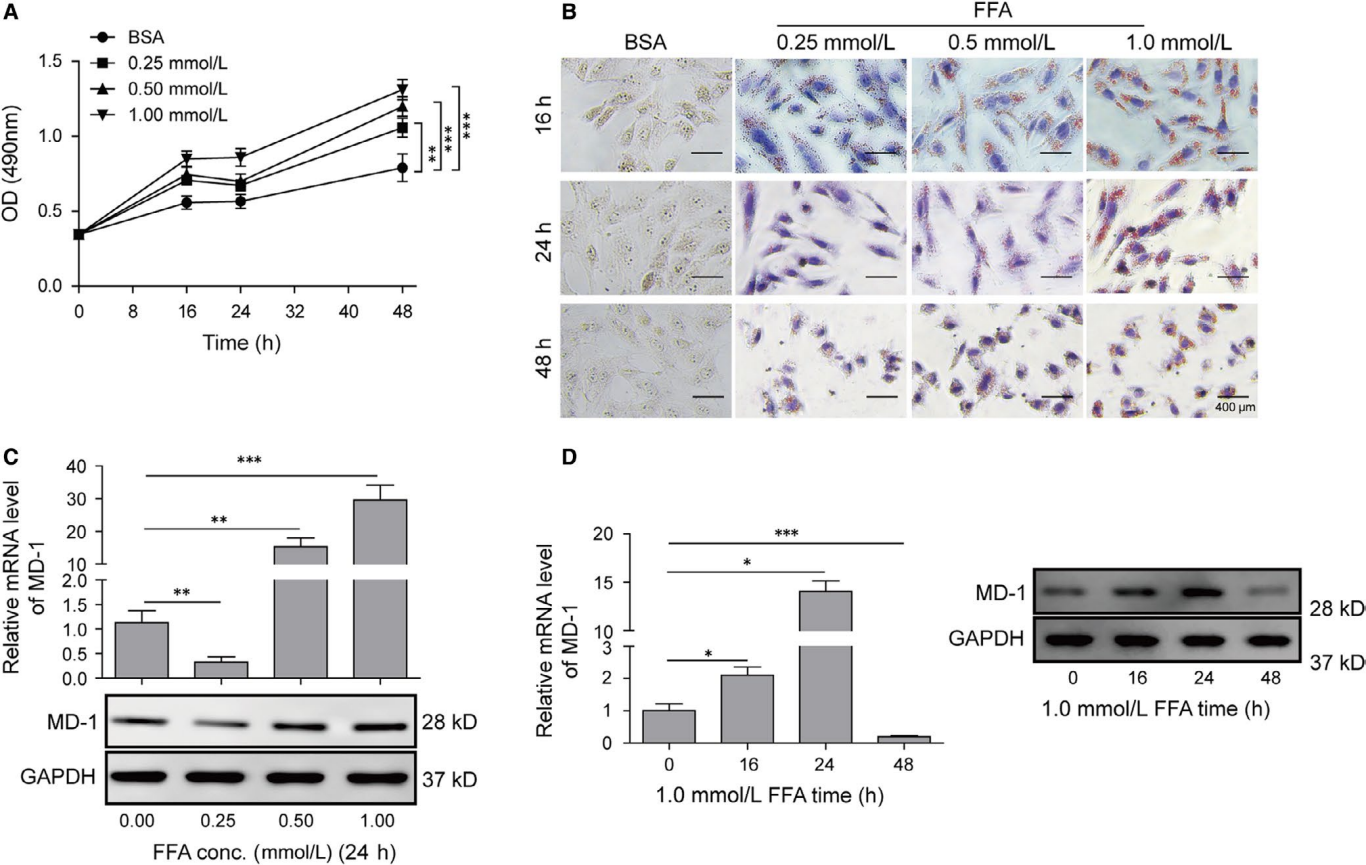
Free fatty acid (FFA) induced lipid accumulation and MD‐1 differential expression in cardiomyocytes. (A) Proliferation curve of H9C2 measured with MTT assay. (B) Oil red O staining of H9C2. Cellular lipid accumulation was stained as bronzing droplets in the images. (C) qPCR and western blot analysis of MD‐1 level. The mRNA and protein levels of MD‐1 were determined following the treatment of FFA at the indicated doses. GAPDH was used as an internal control. (D) qPCR and western blot analysis of MD‐1 level. The mRNA and protein levels of MD‐1 were determined following the treatment of 1.0 mmol/L FFA for the indicated time. *P < 0.05, **P < 0.01, ***P < 0.001

To create a high‐fat induction in vitro, FFA, containing oleic acid and palmitic acid at 2:1 molar ratio, was used to stimulate H9C2 cardiomyocytes with doses of 0.25 mmol/L, 0.5 mmol/L and 1 mmol/L. Results showed that FFA treatment enhanced the cell proliferation in a time‐ and dose‐dependent manner (Figure 1A). By oil red O staining, lipid droplets were observed to present in H9C2 cells with FFA treatment, indicating lipid deposition in high‐fat‐induced cardiomyocytes and increased as dose ascending (0.25, 0.5 and 1 mmol/L) (Figure 1B). However, the morphology of cells changed significantly after FFA stimulation for 48h, suggesting that long‐term high‐fat induction resulted in cytoskeleton damage and pathological remodelling in cardiomyocytes.

We subsequently detected the changes of MD‐1 expression in cardiomyocytes exposed to FFA. The results of Western blot and qPCR revealed that after 24 hours treatment, low dose (0.25 mmol/L) FFA suppressed the expression of MD‐1, which was significantly increased at high dose treatment, like 0.5 mmol/L and 1 mmol/L (Figure 1C). H9C2 cells treated with 1 mmol/L of FFA demonstrated increased MD‐1 level compared to the control, and the expression was especially high in 24 hours treatment; However, dramatic decrease in MD‐1 was observed in cells with the treatment expands to 48 hours, which we suspected that was caused by protein degradation during high dose FFA‐induced cardiomyocytes remodelling (Figure 1D). Therefore, FFA as an inducer of cardiomyocytes injury is involved in promoting lipid accumulation, cell proliferation and MD‐1 may play roles in these processes.

### 3.2 MD‐1 protected cardiomyocytes to resist pathological remodelling induced by FFA stimulation in vitro

**Figure 2:**
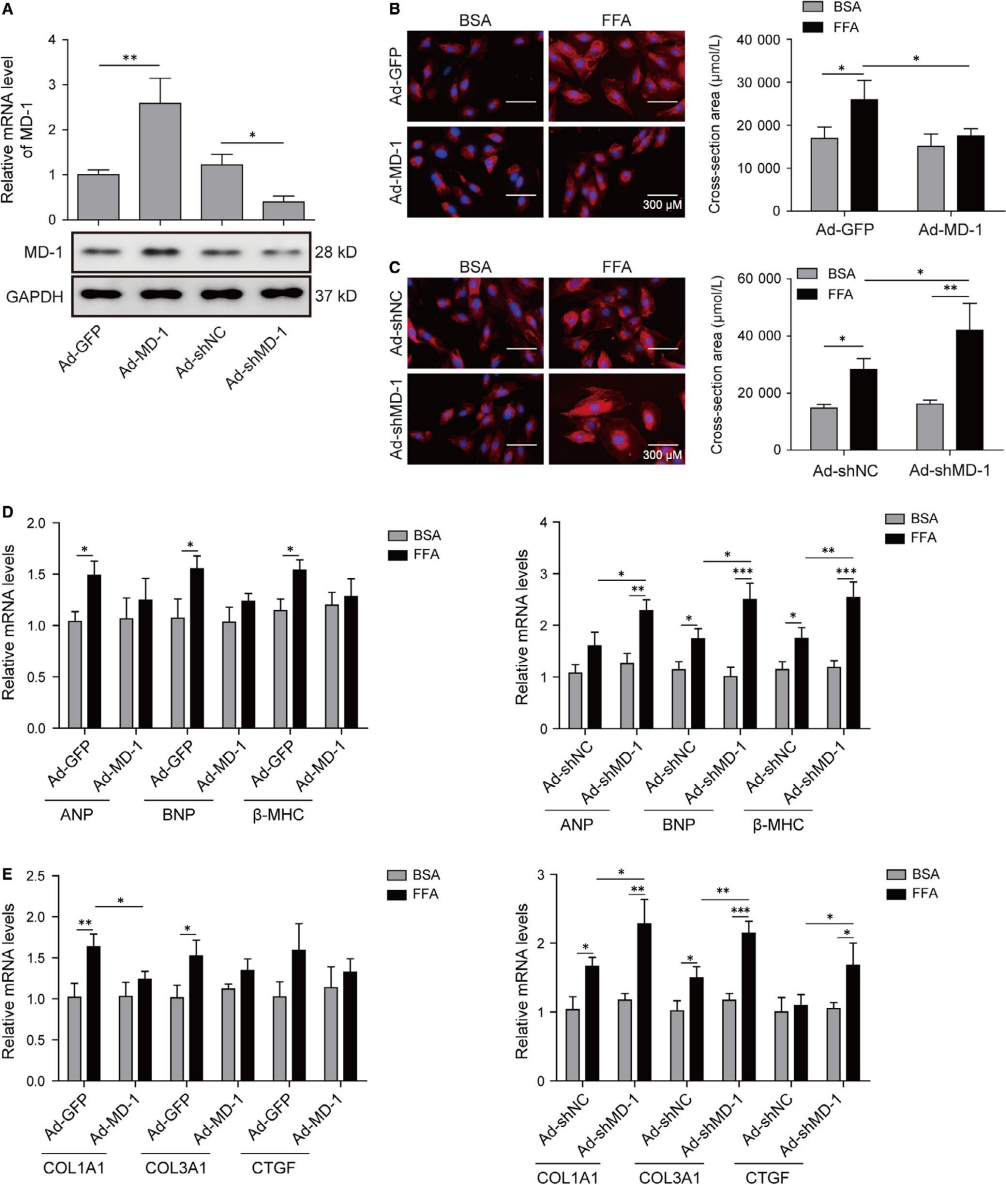
MD‐1 protected cardiomyocytes to resist pathological remodelling induced by free fatty acid (FFA) stimulation in vitro. (A) qPCR and western blot analysis of MD‐1 level in H9C2. The mRNA and protein levels of MD‐1 in H9C2 were determined after cardiomyocytes transfection with MD‐1 overexpression (Ad‐MD‐1) or knockdown (Ad‐shMD‐1) recombinant adenovirus for 48 h. (B) TRITC Phalloidin staining of H9C2 to measure the cross‐section area of cardiomyocytes after adenovirus transfection and FFA treatment for 48 h. (C) qPCR and western blot analysis of ANP, BNP and β‐MHC levels in H9C2 cells as described in B. (D) qPCR and western blot analysis of COL1A1, COL3A1 and CTGF expressions in H9C2 cells as described in B. *P < 0.05, **P < 0.01, ***P < 0.001

To examine the in vitro role of MD‐1 in pathological remodelling, we performed gain‐ and loss‐of‐function studies in H9C2. Cells infected with adenoviruses harbouring the coding sequences (cds) of human MD‐1 (Ad‐MD‐1) or shRNA (Ad‐shMD‐1) for interfered MD‐1 to over‐ or under‐express MD‐1 gene in vitro, and the efficiencies were measured in mRNA and protein levels by qPCR and western blot (Figure 2A). TRITC Phalloidin staining revealed that neither overexpression nor knockdown of MD‐1 altered the cell size of H9C2 cultured under the basal condition (BSA group), however, FFA prominently induced hypertrophy in cardiomyocytes as the cross‐section area of cells increased, which were exacerbated by MD‐1 knockdown but relieved by MD‐1 overexpression (Figure 2B). qPCR and western blot data demonstrated that up‐or down‐regulation of MD‐1 level did not change the mRNA transcription levels of hypertrophy markers including ANP, BNP and β‐MHC, in H9C2 cultured in medium containing BSA; however, silencing of MD‐1 aggravated the transcriptional up‐regulation of these hypertrophy and fibrosis biomarkers in cardiomyocyte exposed to FFA stimulation, which were suppressed through MD‐1 overexpression (Figure 2C,D). Consistent results were obtained in genes COL1A1, COL3A1 and CTGF, known as mediators of fibrosis (Figure 2E,F). These results indicated that MD‐1 protected cardiomyocytes to resist FFA‐induced pathological remodelling in vitro.

### 3.3 Silencing of MD‐1 accelerated high‐fat diet induced myocardial function injury through increased cardiac fibrosis

**Figure 3:**
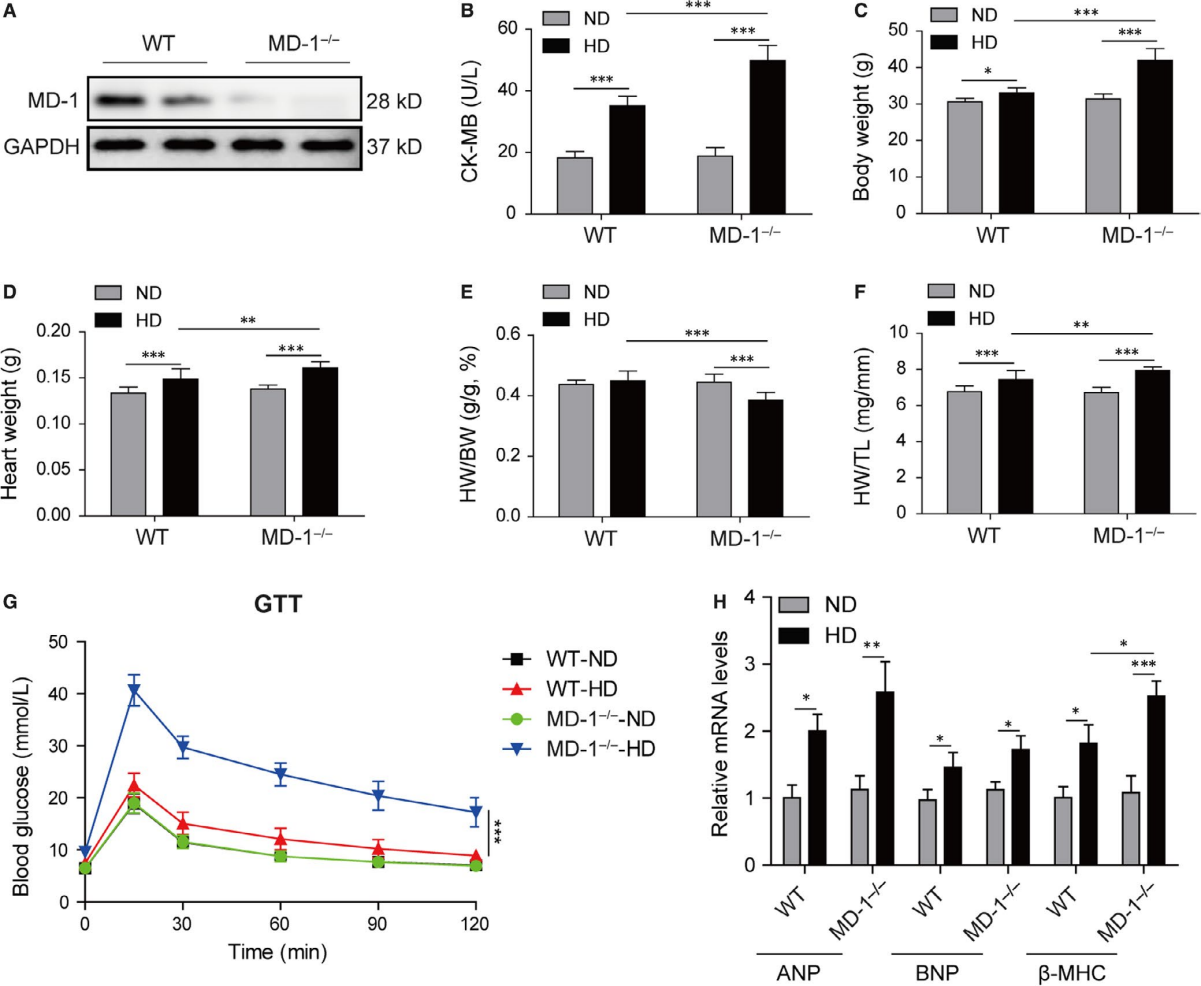
Silencing of MD‐1 promoted high‐fat diet induced myocardial damage. (A) Western blot validated the knockout efficiency in MD‐1^−/−^ mice compared to wild‐type mice (WT). (B) The content of serum creatine kinase‐MB (CK‐MB), a common serum marker for myocardial damage in MD‐1^−/−^ mice and WT mice after high‐fat diet feed for 20 wk (n = 12). (C‐F) BW, HW, HW/BW and HW/TL for the indicated groups (n = 12). (G) Glucose tolerance testing on MD‐1^−/−^ mice and WT mice after high‐fat diet feed for 20 weeks (n = 12). (H) qPCR analysis of hypertrophy markers ANP, BNP and β‐MHC in MD‐1^−/−^ hearts and WT mice hearts after high‐fat diet feed for 20 weeks (n = 4). *P < 0.05, **P < 0.01, ***P < 0.001

To investigate the role of MD‐1 in obesity cardiomyopathy in vivo, MD‐1 knockout mice (MD‐1^−/−^) were fed with high‐fat diet (HD) for 20 weeks. Western blot validated the knockout efficiency in MD‐1^−/−^ mice compared to wild‐type mice (WT), and the MD‐1 protein was barely expressed (Figure 3A). HD induction increased significantly serum lipid indexes including TC, triglyceride and LDL, which were further elevated in MD‐1^−/−^ mice fed with HD (Table 2). The content of CK‐MB, a common serum marker for cardiac damage, was observably elevated after HD induction, especially in MD‐1^−/−^ mice (Figure 3B), indicating that HD‐induced damage in heart tissue, which was much more serious in MD‐1^−/−^ mice. The BW, HW and ratio of HW/TL were raised markedly after HD induction, especially in MD‐1^−/−^ mice (Figure 3C,D,F). However, the HW/BW ratio was lower dramatically in MD‐1^−/−^ mice with HD than WT mice with HD (Figure 3E). GTT also revealed reduced glucose tolerance in MD‐1^−/−^ mice with HD than WT mice with HD (Figure 3G). The mRNA of hypertrophy markers ANP, BNP and β‐MHC were up‐regulated in heart tissues of mice for HD feed, however, there is surprisingly just slight increases in MD‐1^−/−^ mice compared to WT mice after HD feed (Figure 3H).

**Table 2:** Serum lipid indexes measurements in MD‐1 deficient mice (MD‐1^−/−^) compared to wild‐type mice after high‐fat diet (HD) feed for 20 wk

Parameters	WT		MD‐1^−/−^	
	ND	HD	ND	HD
Total cholesterol (mmol/L)	1.933 ± 0.216	4.087 ± 0.412^a^	1.888 ± 0.233	4.813 ± 0.687^a^, ^b^
Triglyceride (mmol/L)	0.592 ± 0.104	1.423 ± 0.175^a^	0.700 ± 0.173	1.897 ± 0.237^a^, ^b^
Low‐density lipoprotein (mmol/L)	0.860 ± 0.121	1.797 ± 0.268^a^	0.784 ± 0.112	1.868 ± 0.190^a^
CK‐MB (U/L)	17.517 ± 2.084	34.933 ± 3.190^a^	18.808 ± 2.742	49.775 ± 4.935^a^, ^b^

a Significant difference of mice feed with high‐fat diet (HD) compared to normal diet (ND).

b Significant difference for HD‐treated MD‐1^−/−^ mice compared to wild‐type one (WT).

**Figure 4:**
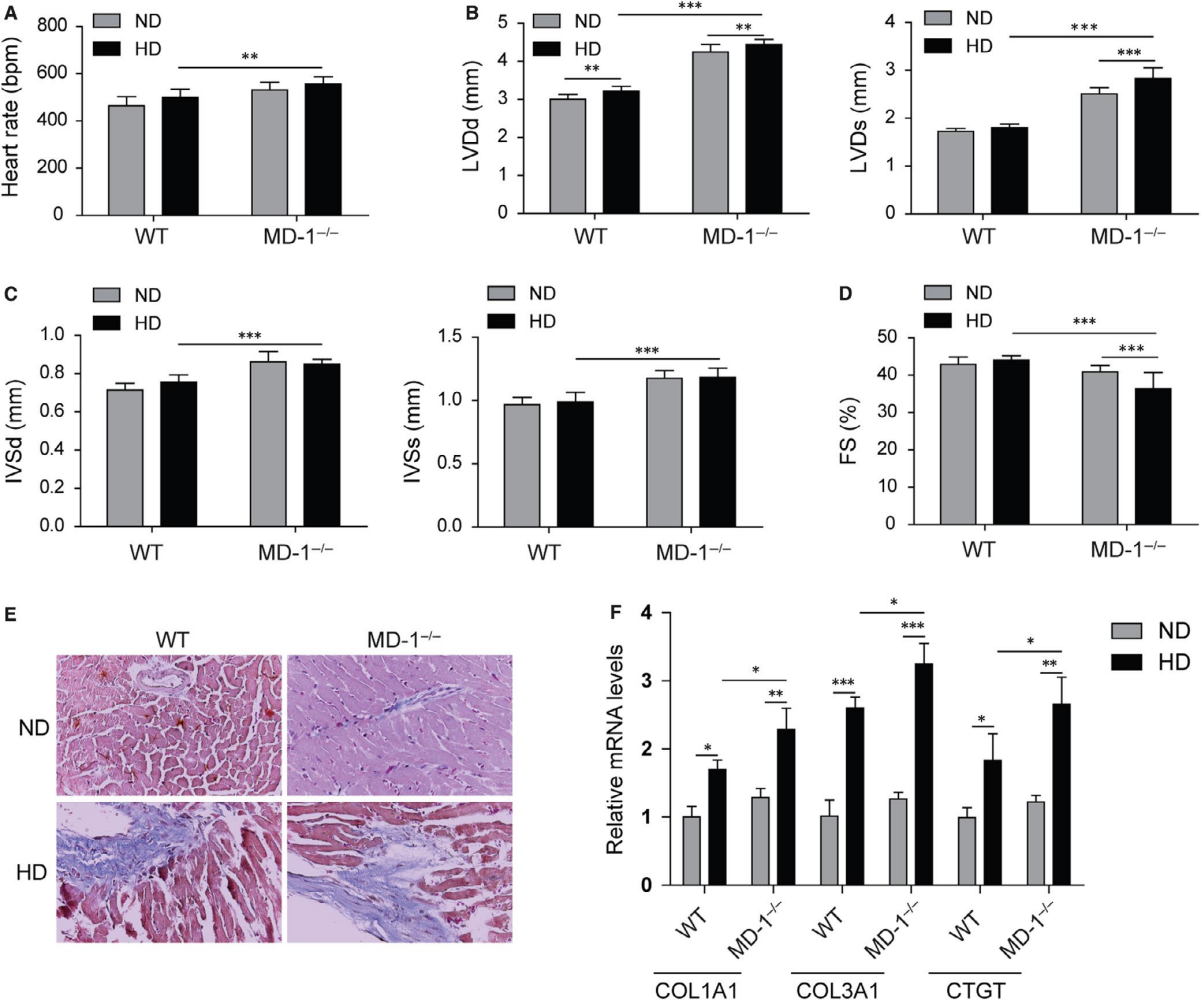
Silencing of MD‐1 accelerated high‐fat diet induced myocardial dysfunction. (A‐D) Echocardiographic results for MD‐1^−/−^ mice and wild‐type (WT) mice after high‐fat diet feed for 20 wk (n = 12). (E) Masson's trichrome staining of heart tissues from MD‐1^−/−^ mice and WT mice after high‐fat diet feed for 20 wk (n = 4). (F) qPCR analysis of fibrosis markers COL1A1, COL3A1 and CTGF in MD‐1^−/−^ hearts and WT mice hearts after high‐fat diet feed for 20 wk (n = 4). *P < 0.05, **P < 0.01, ***P < 0.001

For cardiac function analysis, echocardiographic measurements were performed and the MD‐1^−/−^ mice with HD feed exhibited increased cardiac dilation and dysfunction, including faster HR (Figure 4A), increased LVDd, LVDs, IVSd and IVSs (Figure 4B,C), and reduced FS (Figure 4D) in MD‐1^−/−^ mice than WT mice after HD feed. For further observation of fibrosis, masson's trichrome staining was performed. As expected, increased perivascular and interstitial fibrosis were observed in hearts of mice with HD feed, and these increases were enlarged in hearts of MD‐1^−/−^ mice with HD feed (Figure 4E). The mRNA levels of COL1A1, COL3A1 and CTGF were up‐regulated significantly in MD‐1^−/−^ hearts than normal hearts from WT mice (Figure 4F). All these results indicated that silencing of MD‐1 accelerated myocardial function injury induced by HD feed in vivo through increasing cardiac fibrosis.

### 3.4 Overexpression of MD‐1 alleviated high‐fat diet induced cardiac dysfunction in vivo

**Figure 5:**
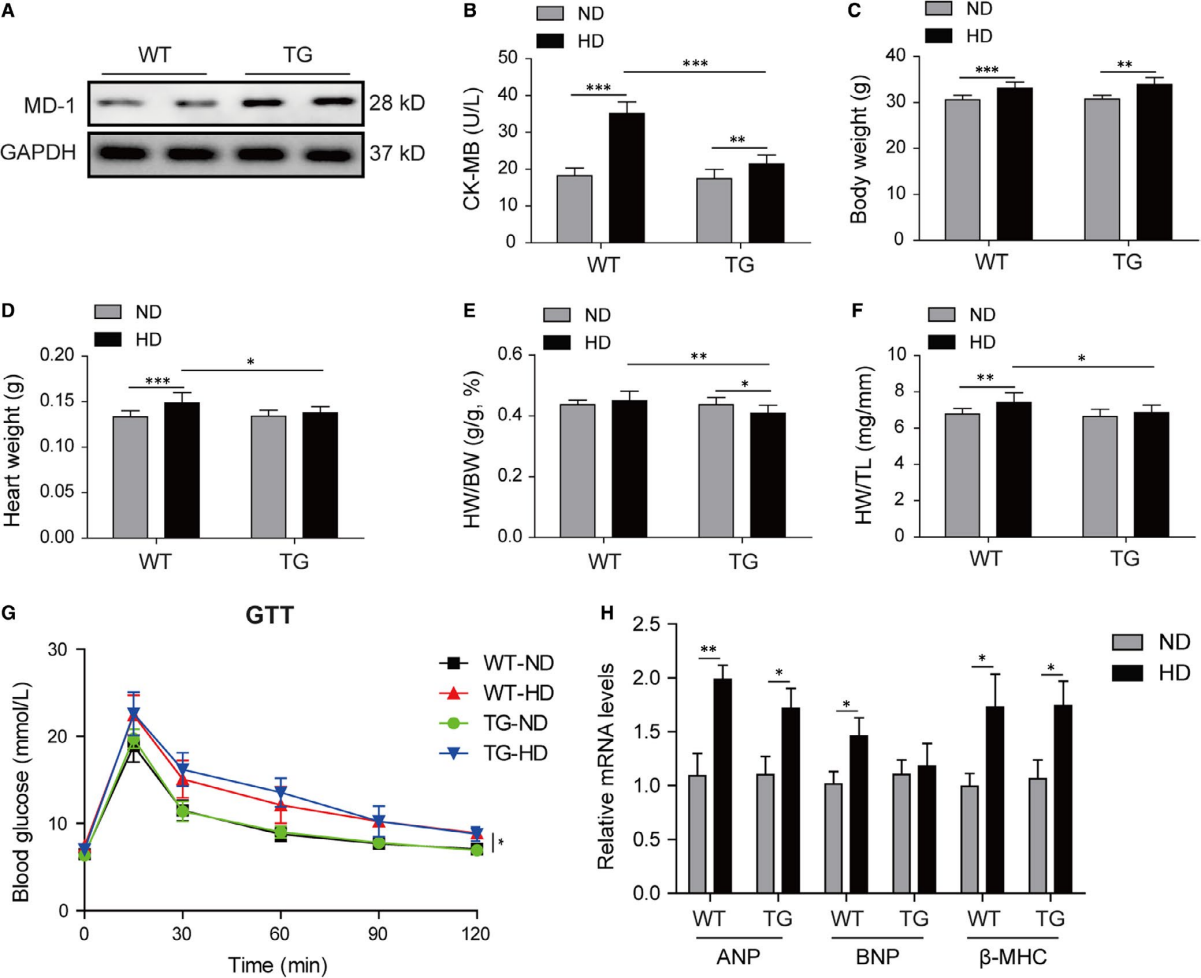
Overexpression of MD‐1 alleviated high‐fat diet induced myocardial damage. (A) Western blot validated the overexpression efficiency in MD‐1 transgenic mice (TG) compared to wild‐type mice (WT). (B) The level of serum CK‐MB in TG and WT mice after high‐fat diet feed for 20 wk (n = 12). (C‐F) BW, HW, HW/BW and HW/TL for the indicated groups (n = 12). (G) Glucose tolerance testing on TG and WT mice after high‐fat diet feed for 20 wk (n = 12). (H) qPCR analysis of hypertrophy markers ANP, BNP and β‐MHC in TG and WT mice hearts after high‐fat diet feed for 20 wk (n = 4). *P < 0.05, **P < 0.01, ***P < 0.001

To further confirm the effects of MD‐1 on heart function in vivo by high‐fat diet feed, transgenic mice overexpressing specially in myocardial cells by the α‐myosin heavy chain (α‐MHC) promoter were generated. Western blot validated that the overexpression efficiency in transgenic mice (TG) compared to wild‐type mice (WT), and the MD‐1 protein level was almost tripled (Figure 5A). The enhanced activity of serum CK‐MB by HD feed was observably suppressed in TG mice than WT mice (Figure 5B), indicating that cardiac damage induced by HD were alleviated by overexpression of MD‐1. The serum lipid indexes for TC, triglyceride and LDL and body weight were elevated remarkedly in mice by HD feed (Table 3 and Figure 5C); however, there was no significant difference between the HD‐treated TG mice and WT mice on these indexed except TC (Table 3 and Figure 5C). The HW, ratios of HW/BW and HW/TL of TG hearts were lower markedly than WT hearts after HD feed (Figure 5D‐F). Slight reduced glucose tolerance was observed in mice with HD treatment, however, there was no difference between TG mice and WT mice (Figure 5G). The increased mRNA levels of hypertrophy markers ANP, BNP and β‐MHC induced by HD were no different in TG hearts compared to the WT mice hearts (Figure 5H).

**Table 3:** Serum lipid indexes measurements in MD‐1 overexpression transgenic mice (TG) compared to wild‐type mice after high‐fat diet (HD) feed for 20 weeks

Parameters	WT		TG	
	ND	HD	ND	HD
Total cholesterol (mmol/L)	1.897 ± 0.564	3.423 ± 0.317^a^	1.849 ± 0.211	4.006 ± 0.309^a^, ^b^
Triglyceride (mmol/L)	0.675 ± 0.100	1.417 ± 0.356^a^	0.668 ± 0.085	1.368 ± 0.343^a^
Low‐density lipoprotein (mmol/L)	0.793 ± 0.282	1.547 ± 0.111^a^	0.794 ± 0.102	1.665 ± 0.263^a^
CK‐MB (U/L)	19.050 ± 1.789	35.383 ± 3.278^a^	17.450 ± 2.481	21.375 ± 2.470^a^, ^b^

a Significant difference of mice feed with HD compared to normal diet (ND).

b Significant difference for HD‐treated TG mice compared to wild‐type one (WT).

**Figure 6:**
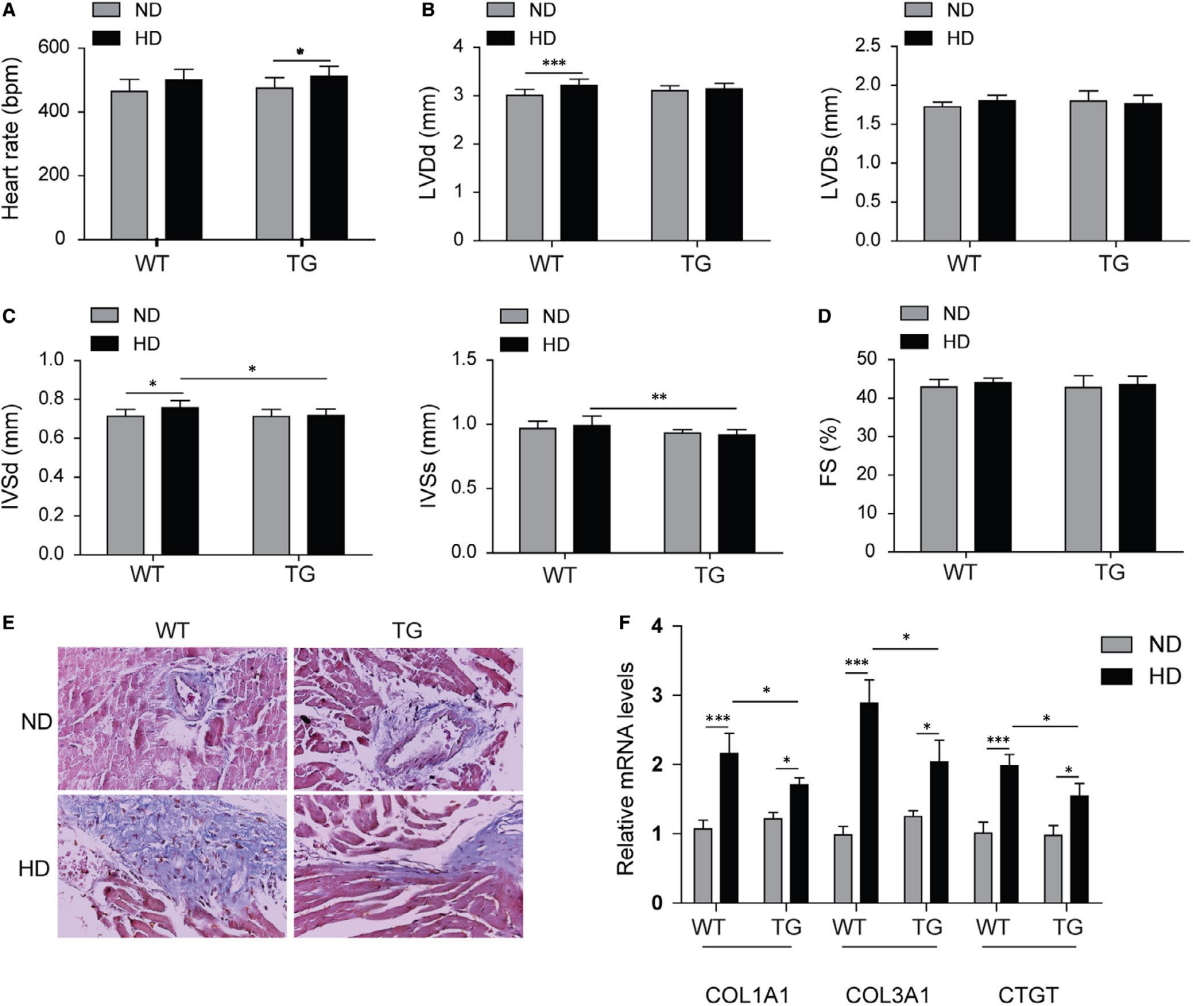
Overexpression of MD‐1 protected myocardial function against high‐fat stimulation. (A‐D) Echocardiographic results for MD‐1 transgenic mice (TG) and wild‐type mice (WT) after high‐fat diet feed for 20 wk (n = 12). (E) Masson's trichrome staining of heart tissues from TG mice and WT mice after high‐fat diet feed for 20 wk (n = 4). (F) qPCR analysis of fibrosis markers COL1A1, COL3A1 and CTGF in TG hearts and WT mice hearts after high‐fat diet feed for 20 wk (n = 4). *P < 0.05, **P < 0.01, ***P < 0.001

For cardiac function analysis, echocardiographic measurements were also performed in TG and WT mice. The heart rate was faster in TG mice after HD feed than ND feed; however, the increase in heart rate in TG mice compared to WT was no significant (Figure 6A). The IVSd and IVSs were decreased in TG hearts than WT hearts after HD feed (Figure 6B,C); however there was no significant difference in LVDd, LVDs or FS in TG hearts (Figure 6D). The increased perivascular and interstitial fibrosis induced by HD feed was reduced remarkedly in TG hearts compared to WT hearts (Figure 6E). Consistently, the mRNA levels of COL1A1, COL3A1 and CTGF were down‐regulated significantly in TG hearts than WT hearts with HD feed (Figure 6F). All these results indicated that overexpression of MD‐1 alleviated the myocardial dysfunction induced by HD feed through inhibiting cardiac fibrosis.

### 3.5 MD‐1 mediated HD‐induced cardiac pathological remodelling via negatively regulating MAPK and NF‐κB signalling

**Figure 7:**
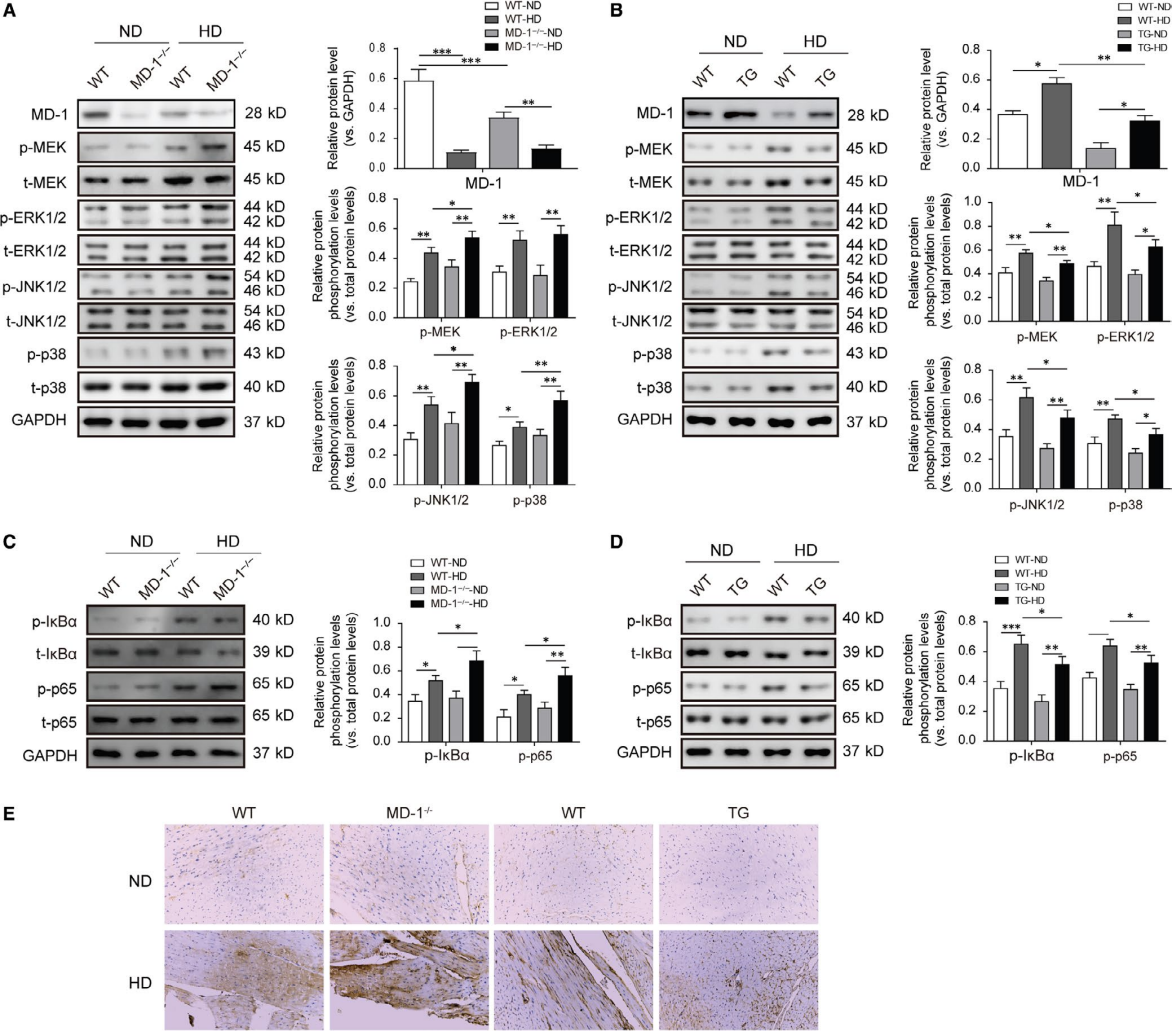
MD‐1 mediated high‐fat diet‐induced cardiac pathological remodelling via negatively regulating MAPK and NF‐κB signalling. (A, C) Western blot analysis for the phosphorylation levels of MEK, ERK, JNK, p38, IκBα and p65, relative to levels of the total one, in MD‐1^−/−^ hearts and wild‐type (WT) mice hearts after high‐fat diet feed for 20 wk. (B, D) Western blot analysis for the phosphorylation levels of MEK, ERK, JNK, p38, IκBα and p65, relative to levels of the total one, in TG hearts and WT mice hearts after high‐fat diet feed for 20 wk. (E, F) Representative images of NF‐κB p65 immunohistochemistry analysis for heart tissues from TG or MD‐1^−/−^ mice models after high‐fat diet feed for 20 wk. *P < 0.05, **P < 0.01, ***P < 0.001

Compared with normal diet, high‐fat diet administration markedly decreased the expression of MD‐1 in WT mice hearts and transgenic mice hearts; however, the effect of HD was less in TG group than in WT group (Figure 7A,B). Since MD‐1 is reported to deliver cardiac protection against cardiac hypertrophy and suppresses cardiac dysfunction during the remodelling process through modulating MEK‐ERK 1/2 and NF‐κB signalling pathways.^19^ Thus, we investigated the expression of MAPK and NF‐κB pathways in the hearts of mice models. Western blot showed that HD significantly increased the relative phosphorylation levels of MEK, ERK, JNK, p38, IκBα and p65 (normalized to their total protein levels), especially in MD‐1^−/−^ hearts (Figure 7A,C); however, these changes were stopped in TG hearts (Figure 7B,D). Consistent results of NF‐κB p65 expression by immunohistochemistry staining were observed in hearts tissues from TG or MD‐1^−/−^ mice models (Figure 7E). These data indicated the MAPK and NF‐κB signalling pathways were activated after HD stimulation or MD‐1 silencing, but that were inhibited by MD‐1 overexpression.

**Figure 8:**
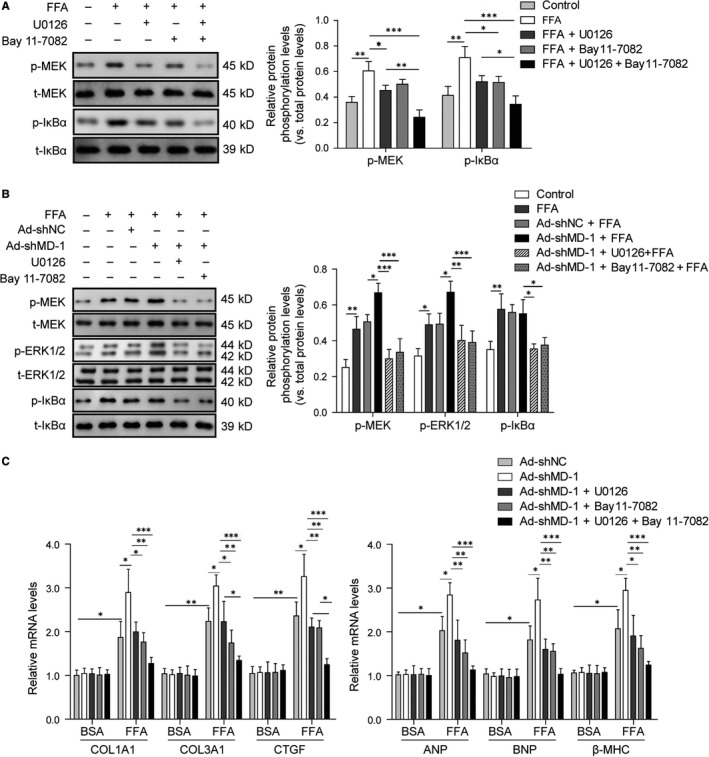
Inactivation of MAPK and NF‐κB signalling pathways by inhibitors rescues the adverse effects of MD‐1 deficiency on high‐fat stimulation induced cardiac remodelling. (A) Western blot analysis for phosphorylation levels of MEK1/2 and IκBα, relative to levels of the total one, in H9C2 cells pre‐treated with 10 μmol/L U0126 and 10 μmol/L Bay 11‐7082 for 30 min before FFA stimulation. (B) Western blot analysis for phosphorylation levels of MEK1/2, ERK1/2 and IκBα, relative to levels of the total one, in MD‐1 deficient H9C2 cells (Ad‐shMD‐1) treated with U0126, Bay 11‐7082 and FFA stimulation. (C) qPCR analysis for the mRNA levels of ANP, BNP, β‐MHC, COL1A1, COL3A1 and CTGF in MD‐1 deficient H9C2 cells (Ad‐shMD‐1) treated with U0126, Bay 11‐7082 and FFA stimulation. *P < 0.05, **P < 0.01, ***P < 0.001

In order to confirm the modulation of MD‐1 on MAPK and NF‐κB signalling, ERK inhibitor U0126 and NF‐κB inhibitor Bay 11‐7082 were used to pre‐treat H9C2 cells transfected with Ad‐shMD‐1 adenovirus before FFA stimulation. The protein phosphorylation levels of MEK and IκBα were elevated after FFA stimulation, which was suppressed remarkedly by U0126 and Bay 11‐7082 respectively (Figure 8A). Silencing of MD‐1 by shRNA adenovirus promoted overactivation of MAPK and NF‐κB signalling pathways, which was suppressed remarkedly by U0126 and Bay 11‐7082 (Figure 8B). The up‐regulated mRNA levels of hypertrophy markers (ANP, BNP and β‐MHC) and fibrosis mediators (COL1A1, COL3A1 and CTGF), induced by MD‐1 silencing and FFA stimulation, were reduced dramatically by U0126 and Bay 11‐7082, partly especially by combined treatment of the two inhibitors (Figure 8C), indicated that the inactivation of MAPK and NF‐κB signalling pathways alleviated the pathological remodelling induced by MD‐1 silencing or FFA stimulation.

To summary, we have revealed a negative modulation pattern of MD‐1 and MAPK/NF‐κB signalling pathways to protect myocardial structure and function in against hyperlipaemia‐induced pathological remodelling, providing novel insights into the mechanisms of obesity cardiomyopathy and targeting MD‐1 may be a feasible strategy for obesity cardiomyopathy in clinical.

## 4 DISCUSSION

Obesity is known to contribute to the development of cardiomyopathy.^3^, ^24^, ^25^, ^26^, ^27^ Although the underlying mechanism has been studies recently, the exact reason of obesity‐induced cardiac hypertrophy and fibrosis is not fully understood. Wang et al reported that chronic high‐fat diet induced myocardial hypertrophy and fibrosis by inhibiting GSK‐3β and up‐regulating the transcription factors yes‐associated protein (YAP1) and nuclear GATA binding protein 4 (GATA4) in HD mice, which induced the transcription expression of hypertrophy‐related genes.^28^ A recent study suggested that disrupted metabolism in early obesity interfering immune responses in cardiomyocytes, resulting in fibrosis and hypertrophy.^27^ In this study, we also proved that FFA induced increased expression of cardiac hypertrophy and fibrosis related genes and proteins. Furthermore, MD‐1 down‐regulated after high‐fat stimulation in vivo and in vitro, indicating that MD‐1 may play important roles in the process of cardiac pathological remodelling induced by high‐fat stimulation.

Secreted glycoprotein MD‐1 is previously reported to regulate immune response through its complex form with RP105 in immune cells, such as B‐cells, dendritic cells and macrophages.^11^, ^15^, ^18^, ^29^ Recently, our group researchers have shown that MD‐1 was also highly expressed in human hearts and it attenuated pressure overload‐induced cardiac fibrosis and hypertrophy through inhibiting MEK‐ERK1/2 and NF‐κB signalling.^21^ Moreover, loss of MD‐1 exacerbates the pressure overload‐induced left ventricular structural and electrical remodelling through hyperactivation of CaMKII signalling and destruction of intracellular Ca^2+^ homeostasis.^30^ Unlike adipose tissue inflammation induced by high‐fat diet in other papers,^14^, ^15^ here we demonstrated that long‐term high‐fat stimulated to reduce of MD‐1 expression in cardiomyocytes in vitro and in vivo, and silencing of MD‐1 exacerbated high‐fat stimulation induced cardiac dysfunction through increasing fibrosis, while overexpression of MD‐1 significantly alleviated these effects of high‐fat stimulation, suggesting that MD‐1 was benefit to protect myocardial function against high‐fat damage.

Here we reported that both NF‐κB and MAPK were activated by high‐fat stimulation in vivo and in vitro, and inhibiting them with corresponding inhibitors ameliorated cardiac hypertrophy and fibrosis in MD‐1 deletion mice. NF‐κB is a critical transcription factor that participates in the TLR4/MD‐2 signalling and fibrosis in diet‐induced obesity,^31^ and its role in cardiac remodelling has been intensively studied.^32^, ^33^, ^34^ Recent evidences suggested that RP105 attenuates ischaemia/reperfusion‐induced myocardial injury via suppressing the activation of the TLR4/NF‐κB signalling pathway.^35^, ^36^ Our research term also have proved that MD1 negatively regulated MEK‐ERK1/2 and NF‐κB signalling pathways and rescued the adverse effects on pressure‐overload induced cardiac remodelling.^21^ Thus, we also check the activities of MAPK and NF‐κB signalling in our study. Results suggested that high‐fat stimulation promoted MEK‐ERK1/2, JNK1/2, p38 and NF‐κB p65 proteins phosphorylation; nevertheless, overexpression of MD‐1 conversely inhibited the activation of these proteins of MAPK and NF‐κB signalling pathways and alleviated cardiac remodelling, form a negative feedback loop. However, whether the MD‐1 expression was mediated by direct NF‐κB transcriptional activation remains to further confirmation.

In conclusion, our study has demonstrated for the first time that MD‐1 play an important function of modulation on cardiac pathological remodelling induced by high‐fat stimulation through negative regulating the MAPK/NF‐κB signalling pathways. Based on these findings, targeting MD‐1 and MAPK/NF‐κB signalling pathways may be feasible strategies for obesity cardiomyopathy in clinical.

## CONFLICT OF INTEREST

The authors declare that they have no competing interests.

## AUTHORS' CONTRIBUTIONS

SC and KB performed the cell culture, high‐fat stimulation and animal experiments, and were major contributors in writing the manuscript. SW performed the adenovirus construction. LY and WG performed the echocardiography and serum lipid index analysis. XM and ZJ performed the histopathological staining analysis. FJ and FH performed the molecular expression detection experiments. JX analysed the data and made the figures. HH designed the experiments, revised the paper and submitted the final versions. All authors read and approved the final manuscript.

## DATA AVAILABILITY STATEMENT

All data generated or analysed during this study are included in this published article [and its supplementary information files].
